# Planning for the evolution of the electric grid with a long-run marginal emission rate

**DOI:** 10.1016/j.isci.2022.103915

**Published:** 2022-02-11

**Authors:** Pieter Gagnon, Wesley Cole

**Affiliations:** 1 National Renewable Energy Laboratory, Golden, CO 80401, USA

**Keywords:** Energy resources, Energy policy, Energy sustainability

## Abstract

Emissions factors are widely used to estimate how various interventions would influence emissions from the electric sector. Both of the most commonly used metrics, however, neglect how changes in electricity demand can influence the structural evolution of the grid (the building and retiring of capital assets, such as generators). This omission can be significant when the factors are intended to comprehensively reflect the consequences of an intervention. In this work we evaluate a lesser known metric—the long-run marginal emission rate (LRMER)—which incorporates both the operational and structural implication of changes in electricity demand. We apply a modeling framework to compare the LRMER to the two near-ubiquitous metrics, and show that the LRMER can outperform the other two metrics at anticipating the emissions induced by a range of interventions. This suggests that adopting the LRMER could improve decision-making, particularly by better capturing the projected role of renewable generators in the evolution of the power sector.

## Highlights

• A long-run marginal emission rate captures both operational and structural impacts
• A LRMER was compared against short-run marginal and average emission rates
• The LRMER outperformed both the SRMER and AER at estimating emission impacts
• Integrating SRMER across an intervention’s lifetime may not describe its total impact

## Graphical abstract

**Figure:**
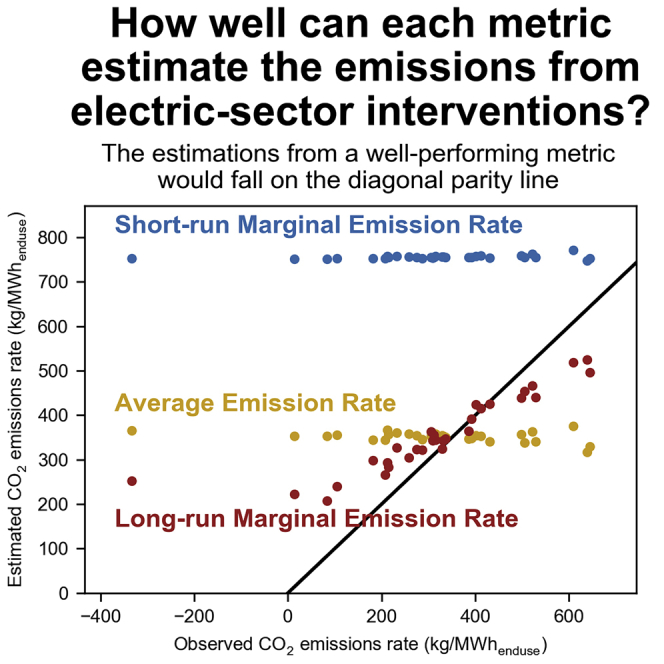

## Introduction

Emissions factors are widely used to estimate what emissions might be induced by new electric loads (or avoided by decreased electrical loads or the construction of new non-emitting generators). ASHRAE standards, LEED credits, the EPA’s Energy Star rating system, state-level building codes, and some corporate clean energy strategies all incorporate emissions factors for guiding decision-making, as just a few prominent examples (ASHRAE, 2020; Google, 2021; New Buildings Institute, 2021; United States Environmental Protection Agency, 2020; Washington State Building Code Council, 2020). They are also used in research to study how interventions such as electric batteries, electric vehicles, solar generators, and energy efficiency measures may affect emissions (Callaway et al., 2017; Graff Zivin et al., 2014; Hittinger and Azevedo, 2015).

The two types of emissions factors that are currently used for nearly all decision-making and research efforts are average emission rates (AER) and short-run marginal emission rates (SRMER).

The AER’s strength is its simplicity: It is derived by dividing the total emissions by the total electricity generation and adjusting for losses (Azevedo et al., 2020; eGrid, 2021). However, when used to estimate the consequences of an intervention it has a well-understood flaw in that changes to a system act on its margin, not its average. The generation mixture induced by new load often looks very different than the current average generation mixture.

The SRMER, on the other hand, is derived by identifying which generator would have supplied more energy if additional load was placed on a grid at a particular point in time (Azevedo et al., 2020; Deetjen and Azevedo, 2019; Gagnon et al., 2020; Siler-Evans et al., 2012; Thind et al., 2017). Often simply referred to as the “marginal emissions rate”, we follow previous literature in adding the “short-run” prefix to make an important distinction more salient: The SRMER is strictly an operational metric, representing how new load would be served *from the existing grid*, while neglecting any influence that new load could have on the structure of the grid (e.g., building or retiring of capital assets such as generators or transmission lines).

Although these two metrics can be suitable for many purposes—such as characterizing the near-term impacts of decisions or describing the current state of the grid through emissions inventories—their neglect of how a change in electric demand may influence the structural evolution of the grid can be a crucial omission if they are being used to comprehensively characterize the consequences of an intervention—especially a long-lived intervention such as a home retrofit, vehicular electrification, energy efficiency measures, or proposed renewable generators. The most important manifestation of this omission is straightforward: Adding electrical load has the potential to induce the construction of more non-emitting generators, such as wind and solar. A metric that is intended to be comprehensive would need to capture this phenomenon.

This leads us to the third metric we will evaluate in this paper: the long-run marginal emission rate (LRMER). Unlike the SRMER—which treats the structure of the grid as fixed—the LRMER is derived from forward-looking projections and explicitly takes into account how changes in load can induce both operational and structural changes to the grid. See Table 1 for metric definitions. Previous literature has introduced this metric (Hawkes, 2014), and here we build on that work by using a modeling framework to show how an LRMER can outperform both an AER and SRMER at estimating the emissions induced over the full lifetime of a load intervention.

**Table 1:** Definitions for the three metrics considered in this work

Metric	Definition
Average emission rate (AER)	Total emissions divided by total electricity consumption.
Short-run marginal emission rate (SRMER)	The emissions per unit change in electricity consumption, where the structure of the electrical grid (e.g., the generation, transmission, and distribution assets) is considered fixed.
Long-run marginal emission rate (LRMER)	The emissions per unit change in electricity consumption, where the influence of the change in demand on both the operation and structure of the grid is taken into account.

Of particular importance, we also show how there is a strong diurnal trend created by the projected contribution of solar energy in the evolution of the US electric grid—i.e., adding load during daylight hours is projected to induce significantly less emissions than adding load during non-daylight hours. We show how the LRMER can anticipate that phenomenon, whereas AER and SRMER do not. This finding suggests that adopting the LRMER metric can represent the projected contribution of solar energy in planning and evaluations in a way that is not currently accomplished through AER or SRMER metrics.

In this article, we present evidence that an LRMER metric can outperform both AER and SRMER metrics for comprehensively evaluating electric-sector interventions. To enable practitioners to act on this, an associated effort has made LRMER projections available for the contiguous US (www.nrel.gov/analysis/cambium.html). We plan to update and re-release this data annually.

## Results

### Comparing the performance of the three metrics

In this section, we evaluate the performance of an LRMER metric and compare it against the near-ubiquitous SRMER and AER metrics.

This effort required projecting the evolution of the US electric grid. For this, we used the ReEDS capacity expansion model, which is a publicly available capacity expansion model that was built to project the evolution and operation of the electric sector in the contiguous US (Brown et al., 2020). We replicated the assumptions from the Mid-case of the National Renewable Energy Laboratory’s 2020 Standard Scenarios (Cole et al., 2020). The Mid-case is a business-as-usual style projection with “reference” fuel prices from the Annual Energy Outlook (EIA, 2020), “moderate” technology cost improvements from the Annual Technology Baseline (NREL, 2020), and representations of state policies enacted when the study was conducted. See Figure S1 for generation and capacity projections.

Then, to evaluate each of the three metrics, we defined a set of 31 different load shapes, and used each metric (calculated from the projected future described above) to estimate the emissions that would be induced if that load were added to the US electric grid. Next, we projected the evolution and operation of the grid over a 20-year period with each of the interventions directly represented in the ReEDS model, and calculated the emissions that each intervention had induced by comparing against the original no-intervention projection. Lastly, we compared each metric’s estimations against the emissions we saw when we directly modeled the interventions—a well-performing metric would be able to accurately anticipate the modeled results.

The load interventions that we created varied in what time-of-day they added load (see Table 2 in the STAR Methods section)—i.e., some added load predominantly during the day, some predominantly during the night, some across all hours, and so forth. The time-varying shapes are of particular importance to our investigation—we are interested in identifying which metric best captures diurnal trends in emission rates (e.g., which metric can best anticipate the difference between adding load during the daytime versus nighttime—as you might see with different operating strategies for electric water heaters or when comparing energy efficiency measures between the residential and commercial sectors).

**Figure 1:**
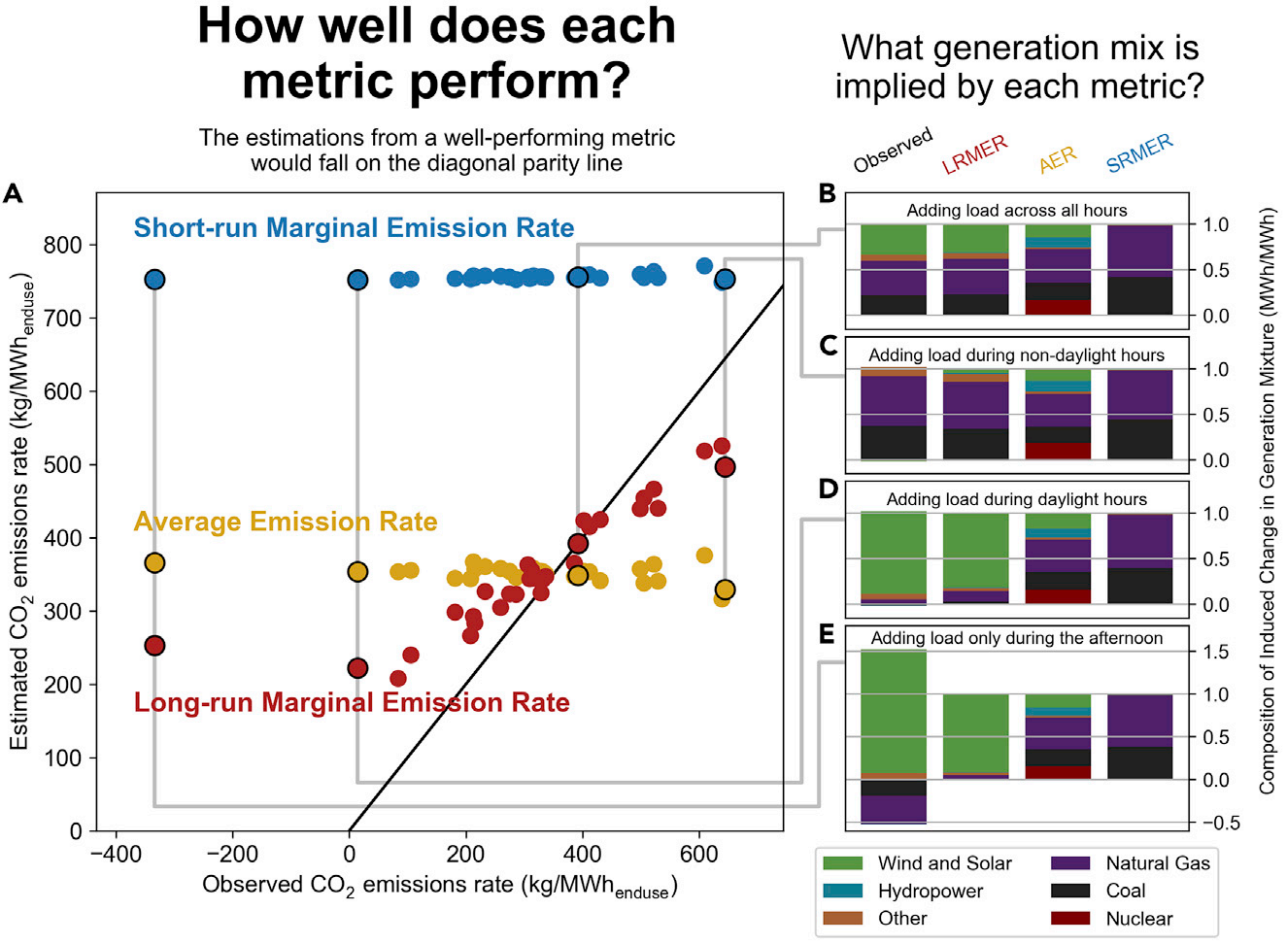
Metric performance and implied generation mixtures (A) parity plot showing the performance of the three metrics. (B–E), observed generation mixture compared against the generation mixtures implied by each metric for four different load shapes: Adding load across all hours, predominantly during non-daylight hours, predominantly during daylight hours, and predominantly during the afternoon. Observed emissions are evaluated for a 20-year period. The LRMER metric is derived from the same 20-year period whereas SRMER and AER metrics are derived from the state of the grid in the year the load was introduced (2024). The root-mean-square errors of the metrics are 131 (LRMER), 201 (AER), and 476 (SRMER).

The parity plot in panel A of Figure 1 shows the performance of each of the three metrics for the 31 different load interventions applied to the contiguous US, starting in 2024 and evaluated for 20 years. If a metric performed well, its points would fall on the diagonal black line, meaning that the metric had exactly anticipated what emissions each load intervention would induce when we implemented that intervention into the capacity expansion model.

Panels B through E of Figure 1 show generation mixtures for four illustrative load interventions (corresponding to load shapes 1, 28, 7, and 2, as given in Table 2), and are meant to help interpret the results in panel A. The left-most bar (“observed”) is the composition of the changes in the generation mixture between the with-intervention and without-intervention model runs, normalized by the total net change in generation, with negative values for generation types that decreased as a result of the intervention. The following three bars then show the mixture that each metric’s method implicitly assumes—the AER mixture, for example, is calculated by adding together each hour’s generation stack weighted by that intervention’s load in each hour, and then normalizing the result (see STAR Methods section).

The observed emissions in Figure 1 are evaluated for a 20-year period. For this first discussion, the LRMER metric is derived from the same 20-year period, but the SRMER and AER metrics are derived from the state of the grid in the year the load was introduced. We do this because standard practice is to likewise derive SRMER and AER metrics from the current state of the grid, not forward-looking projections. We are, therefore, comparing the proposed LRMER metric against the approach currently employed by practitioners. In the following section we compare the performance of the LRMER metric against the SRMER and AER metrics when they also are calculated from projected 20-year periods.

First, we see that the observed emission rates of the 31 load interventions varied significantly—as high as 644 kg/MWh, most between 200 and 500 kg/MWh, several lower than 200 kg/MWh, and a single intervention that actually reduced emissions. Generally, interventions with more load during daylight hours had lower emissions, whereas interventions with more load during the evenings and nighttime had higher emissions (see Table S1 for the data underlying the parity plot).

Looking first at the SRMER points (blue) in panel A of Figure 1, we see that the SRMER metric overpredicted the emissions induced by all the load interventions we examined, often quite significantly (RMSE of 476). This is because the SRMER does not capture how the new load is projected to induce new builds of wind and solar generators, nor does it anticipate the evolution of the grid over the lifetime of the intervention. Rather, it only reflects what the emissions would be if the load was served by the existing fleet of generators. We see this illustrated by looking at the fuel mixtures in the right-most column of panels B through E of Figure 1: because the SRMER treated the grid as fixed, it implied that additional load would be served overwhelmingly by a mixture of natural gas and coal generation, with a relatively small contribution from other fuels. When we compare those mixtures against the observed mixtures (left-most column), however, we see that additional wind and solar generation was often induced by the new load, which cannot be predicted without accounting for structural change.

Importantly, we see that at least for the circumstances we are modeling (the contiguous US, with loads being introduced in 2024, and under the particular grid evolution being projected), the SRMER did not anticipate the differences between adding load at different points in the day. While there were some local diurnal trends in SRMER rates (examples are given in Figure S2), the predictive ability of these trends appears to be poor, at least when assessed as a group as we are doing here. SRMER was most severely incorrect when load was predominantly added during daylight hours (when new load induces a relatively large amount of new solar builds, such as panels D and E), and the closest to being correct when load was added predominantly during the night (when additional load induces very little structural change, such as panel C).

Moving to AER (the yellow points in Figure 1), we see that its estimations tended to be closer to the observed values than the SRMER estimations were (RMSE of 201). Looking at the fuel mixtures in panels B-E, however, we see that this was just happenstance for the particular circumstances we modeled. The AER incorrectly assumed that new load will be served in part by hydropower and nuclear generation while underestimating the share of wind and solar, two countervailing errors that approximately canceled each other out. Therefore, while the error of the AER’s predictions could be low for this particular situation, we stress that that is not a generalizable conclusion, as the AER metric could be significantly off for different countries, specific states within the US at different points in time, and under different assumptions about the future. Notably, however, in a hypothetical condition where the generation mixture that serves new load closely resembles the generation mixture of the existing grid, the AER could become an excellent predictor of the emissions induced by an intervention, with the attractive attribute of being calculatable with empirical data.

Similar to SRMER, the AER metric also did not do a good job of predicting the variation in emissions across different load shapes—there is actually a slight negative correlation between the AER’s prediction and the observed emission rate. This is because the nighttime hours have a relatively higher proportion of hydropower and nuclear generation and the daytime hours have a higher fraction of lower-efficiency fossil generation, resulting in the AER metric incorrectly predicting that nighttime interventions would induce fewer emission than daytime interventions.

Lastly, we look at the LRMER (the red points in Figure 1, RMSE of 131). Because it incorporates how changes to load can induce both operational and structural changes to the grid, it was better able to anticipate how new load could prompt more renewable resources to be built, anticipate the increased utilization of existing resources (e.g., coal), and leave out technologies that are not currently projected to contribute relatively large amounts to incremental generation (e.g., hydropower and nuclear). Because it can capture these forward-looking trends, the LRMER metric outperformed both the AER and SRMER at estimating the emissions induced by changes in load.

Importantly, for the interventions we explored here, the hourly LRMER was able to partially anticipate the differences between the emissions from different load shapes—e.g., it was able to anticipate that adding load in the daytime would generally induce less emissions then adding load during the night. This is because the LRMER, by explicitly incorporating the possibility of load to induce structural change, contained a diurnal trend stemming from the potential for new solar builds. Other variable renewable technologies—such as wind generation—have less prominent diurnal trends, and therefore their contribution is captured by generally lower emission rates across all load shapes. As discussed more fully in the discussion section, this shows us that the LRMER represents an opportunity to incorporate the low-carbon benefits of daytime electricity consumption in a wide swath of planning decisions. This opportunity is more difficult to act on if AER or SRMER metrics are used for planning.

On the two extremes of observed emission rates, the LRMER did not perform quite as well, especially at the lowest end. The LRMER tended to overestimate the emissions induced by mostly daytime interventions and underestimated the emissions induced by mostly nighttime interventions. This is caused, in part, by an interdependency of hours that is difficult to capture in a metric designed to be used across a wide variety of possible shapes. For the daytime loads (exemplified with panels D and E), the extra demand during hours ideal for solar predictably induce solar builds, which then have spillover effects in other hours beyond the ones in which load is being added. The other extreme is an inversion of the same situation: The LRMER anticipated that load added in the evening and nighttime hours would induce some renewable generation (predominantly wind generation, although solar as well if the load starts in the early evening or extends into the morning), but when load was only added during those hours, it was not sufficient to induce new renewable generation investment, resulting in an under-prediction by the LRMER metric.

### Levelized AER and SRMER metrics

As mentioned previously, the metric evaluation shown in Figure 1 used AER and SRMER values from the single year the load was introduced. We chose this to characterize the performance of those metrics when they are calculated from data near the time of deployment, as is common in practice.

**Figure 2:**
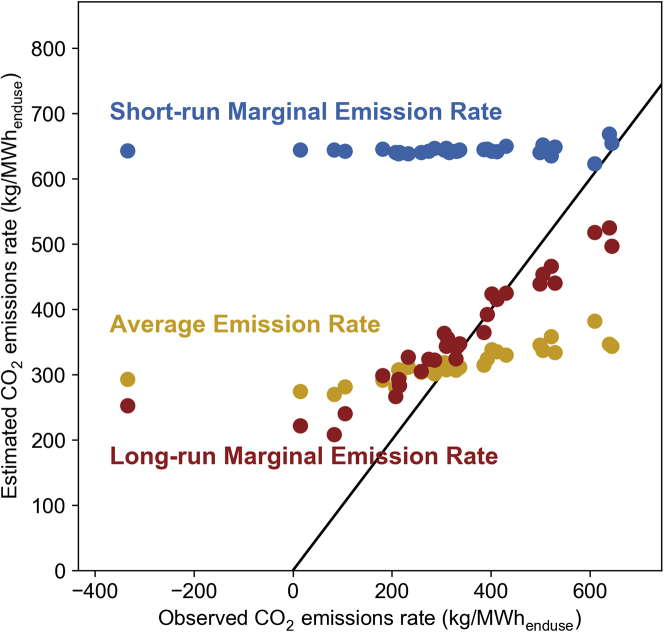
Metric performance when all three metrics are calculated as 20-year levelized values The root-mean-square errors of the metrics are 131 (LRMER), 174 (AER), and 376 (SRMER).

A reader might reasonably wonder, however, if AER and SRMER would perform better if they were also calculated from projected data that covered the lifetime of the intervention. To investigate this, Figure 2 shows the same metric evaluation as Figure 1, but with AER and SRMER calculated from the same 20-year projection that was used to assess the LRMER.

By comparing Figures 1 and 2, we can see that for the conditions modeled here calculating AER and SRMER from projected data did indeed improve their performance. AER’s RMSE decreased from 201 to 174 and SRMER’s decreased from 476 to 376, compared to the 131 of LRMER. As a levelized metric, AER adopted a slight positive correlation with the observed emission rates, although not as great as LRMER.

Because the frequency of coal generators being on the short-run margin decreased in these projected futures, the levelized SRMER values were lower than the ones calculated just from the first-year values, which improves the metric’s performance. The levelized SRMER still performed poorly at anticipating the differences in emission rates from different interventions, however.

Overall, Figure 2 illustrates an important point: the deficiencies of AER and SRMER (when intended to comprehensively capture the emissions impact of interventions) are not only that they fail to anticipate how the grid is projected to evolve over the lifetime of an intervention. Even when they were calculated from the same projections used to calculate the LRMER, they performed worse than it. This is because neither captures how a marginal change in load would further influence the structure of the grid, beyond the already-anticipated evolution. This point is particularly relevant for SRMER—Figure 2 demonstrates that the impact of an intervention is not simply the SRMER summed over the lifetime of the intervention. Even an SRMER calculated from a projected future grid still does not incorporate the impact of structural changes induced by the changes in load, and is therefore incomplete.

### Visualizing the metrics

**Figure 3:**
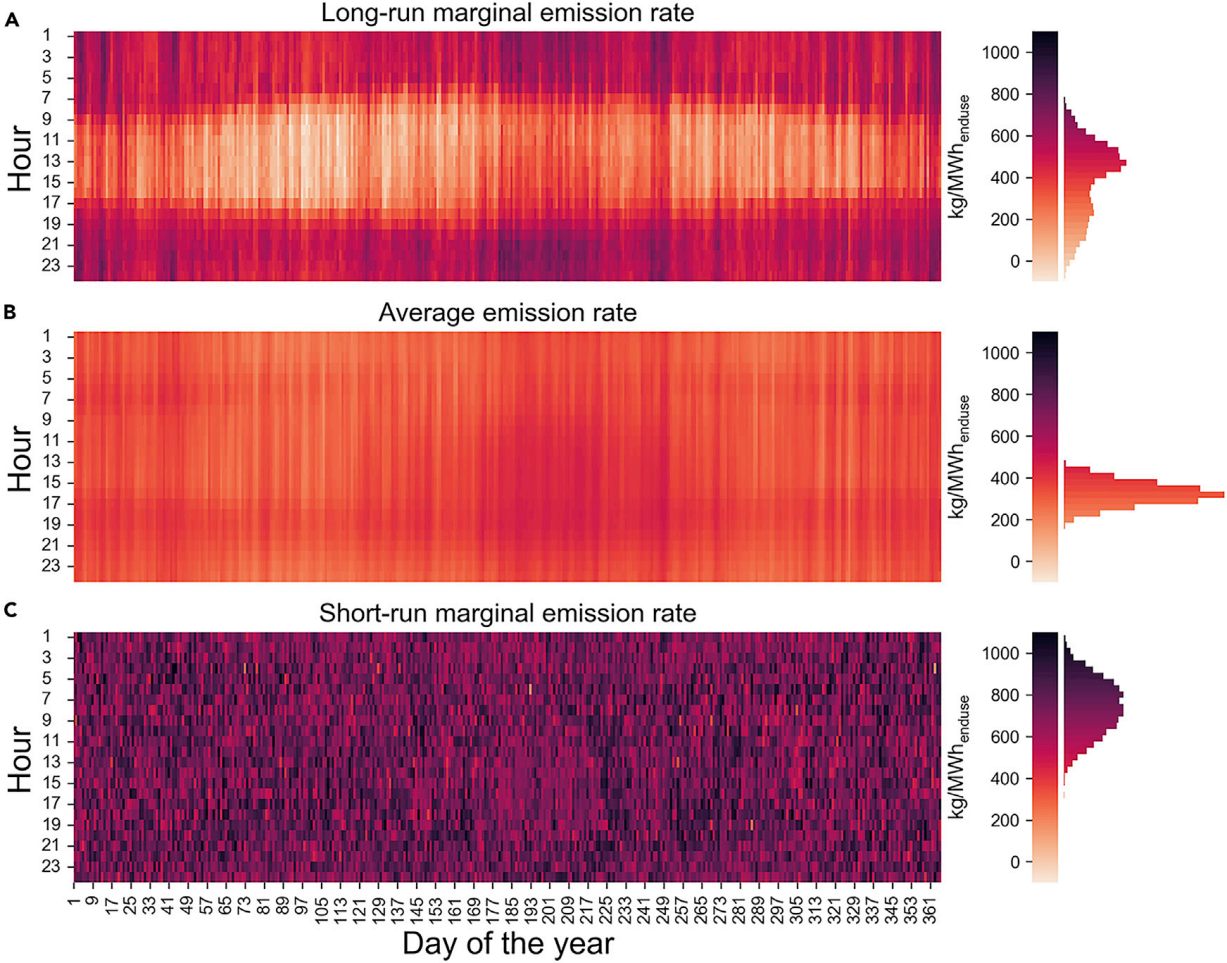
Hourly visualization of the three metrics (A) LRMER. (B) AER. (C) SRMER. CO_2_ from combustion per MWh of end-use load for the contiguous US. Central time zone. LRMER are 20-year levelized values, SRMER and AER are single-year values from 2024.

To build intuition, in Figure 3 we show the hourly values of the metrics. These are the values underlying the metric performance evaluation from Figure 1.

The most prominent observation is that the LRMER is the only one of the three metrics with strong diurnal trends. The relatively low values during daylight hours reflect that increases in electricity consumption during those times are projected to induce new solar capacity, while the contribution of new wind capacity is represented as generally lower emission rates across all hours.

The AER does have faint diurnal trends and relatively stronger seasonal trends. As we saw in the previous section, the diurnal trend is actually counter-predictive, however. Because the share of generation from nuclear, hydropower, and existing wind generators is relatively higher during the night, for example, the AER suggests that predominantly nighttime loads will induce fewer emissions than predominantly daytime loads, which is the opposite of what actually occurs when the long-term interventions are introduced.

In some regions within the US there are seasonal and diurnal trends in SRMER, but we do not observe any in Figure 3. This is primarily because any such local trends are obscured when the SRMER is aggregated to the nation as a whole for visualization. In Figure S2 we provide examples of regions with local trends. Note that whatever predictive ability such regional trends had was embodied in the aggregated SRMER performance shown previously in Figures 1 and 2. This suggests that, at least taken together as a group, they had limited predictive ability.

## Discussion

We saw that by explicitly incorporating how changes in load could induce both operational and structural changes in the grid, the LRMER metric was able to better estimate changes in emissions resulting from a change in end-use electrical load, relative to the AER or SRMER metrics. This suggests that LRMER may be more suitable than AER or SRMER for comprehensive analyses of electric-sector interventions, when those interventions have the potential to influence structural decision-making.

The question of whether an intervention has the potential to influence structural decision-making depends on the characteristics of the intervention and the particular decision-making processes of the resource planners in the relevant region. It is likely that interventions lasting 5 years or longer would be sufficient to warrant using long-run metrics for most locations in the US, given the duration of most planning cycles, although this specific question has not been studied to our knowledge. Shorter-duration or one-off decisions (such as the real-time dispatch of datacenter loads or when to charge an electric vehicle) may still have the potential to influence the structural evolution of the grid, however, particularly if they are part of an ongoing pattern of short-term decisions, and therefore may still warrant the use of a long-run factor (or a new metric that combines near-term operational impacts with long-term structural impacts).

Using metrics that neglect structural change may lead to erroneous conclusions—for example, when considering electrifying end-uses (such as vehicles or water heaters), the SRMER would imply that the new electrified load would largely be met by coal and natural gas. The LRMER, however, would anticipate that the new load would likely prompt new wind and solar generators to be built, resulting in lower induced emissions. Looking at the scenario where we added load across all hours, LRMER projected an emission rate of 392 kg/MWh whereas the SRMER projected an emission rate of 756 kg/MWh—nearly twice as high. This difference may be sufficient to change a key conclusion: whether electrification, on net, reduces or raises total emissions.

The forward-looking perspective of the LRMER was also found to be important for anticipating the implications of adding load during different times of the day—specifically, that load added during daylight hours is projected to induce less emissions than load added during the evenings and nighttime, given the role that solar energy is expected to play in the evolution of the US electric grid. Consider, for example, the emissions induced by different charging strategies of electric vehicles. If we compare a predominantly nighttime charging strategy with a predominantly daytime charging strategy, this work suggests that the LRMER metric would have correctly predicted that daytime charging would induce solar to be built, and consequentially result in lower emissions than nighttime charging. The AER and SRMER would not have anticipated this phenomenon.

Although this paper exclusively modeled end-use load interventions (i.e., demand-side interventions), the concepts may be applicable to decisions on the supply side as well, where the supply side intervention influences the rest of the grid in a similar way as the demand-side interventions we studied here. For example, LRMER metrics may be used to estimate how proposed variable renewable generators, such as wind or solar, would impact emissions, as such an intervention would be similar to decreasing the demand on the rest of the generation fleet. While the LRMER factors suitable for end-use and supply side analyses may be identical in some circumstances, they also may be different under other conditions. One example is when a binding grid-relevant policy is based on retail sales—as is common for renewable portfolio standards—in which case the effects of a change in end-use load may not be equivalent to a change in the injection of busbar-level generation.

### Limitations of the study

This paper gave examples and shows the mechanics of how, under certain assumptions about the future and in a modeling environment, an LRMER metric was able to outperform both the SRMER and AER metrics. Nonetheless, this paper is not an exhaustive exploration of the topic, and we discuss its limitations here.

First, we performed this study using a business-*as*-usual style projection of the future (see model and scenario description methods section). While the mechanics explored here do not change across different futures (e.g., whether or not a metric incorporates the structural consequences of an intervention does not depend on how the future unfolds), the relative performance of the three metrics could be different. A future with less PV could have weaker diurnal trends, degrading the relative improvement of LRMER metric, for example. Alternatively, a future with more rapid power sector decarbonization could have the forward-looking LRMER increase its performance beyond what was seen here. While this paper was intended as a focused discussion around the mechanics of the performance of different emission factors, and therefore did not explore alternative futures, we point interested readers to the ongoing Cambium project (https://www.nrel.gov/analysis/cambium.html) at the National Renewable Energy Laboratory, which has produced emission metrics across a range of different futures.

Relatedly, this paper only analyzed the performance of the three metrics in situations where the future unfolded as was projected when the hypothetical interventions were introduced. All three metrics would be susceptible to general uncertainty about the future. For example, if the cost of solar increased dramatically over the next few years, an LRMER that had anticipated that incremental daytime electricity demand would induce more solar might be erroneous. The AER and SRMER, on the other hand, implicitly assume that the future will look like the past, which does not shield them from variations in their error depending on how the future unfolds—such as significant overestimates of emissions for long-lived interventions if the electric grid continues to decarbonize. We leave this type of uncertainty unexplored in this paper.

We also only performed this analysis for the contiguous US. Later analyses could examine the same topic for other countries or for finer geographic regions within the US. Geographically decomposing nation-wide simulations into fine geographic regions is an ongoing area of research not explored here. The author’s work on this topic is described in the report *Cambium Documentation*: *Version 2021* (Gagnon et al., 2021), with further updates expected to be made available through the aforementioned Cambium project.

Next, a significant weakness of an LRMER is that its derivation requires projections of how the electric grid will evolve, and is therefore only as good as those projections. An LRMER based on a poorly built model could produce worse estimates then AER or SRMER metrics derived from empirical methods.

Lastly, in this work we implicitly assumed that the load interventions were anticipated and planned for, and therefore the grid’s structure adapted immediately when the load interventions were introduced—this might be reasonable for an intervention that is anticipated by a resource planner (such as the load reductions achieved through a large energy efficiency campaign), but an unexpected load addition might take several years to influence the structure of the grid. This suggests that for interventions that are not anticipated by resource planners, it may be appropriate to develop emissions factors from models that explicitly represent the time lag in structural change, or by approximating that phenomenon by creating a blended metric that is composed of both SRMER (weighted by the length of time until the intervention is expected to influence structural decisions) and LRMER factors (weighted by the remaining lifetime of the intervention). Creating such a blended metric would also give an opportunity to take advantage of high-quality empirical data for near-term emission rate estimates, switching to modeled data further out.

## STAR★Methods

### Key resources table

**Table:** 

REAGENT or RESOURCE	SOURCE	IDENTIFIER
**Deposited data**		

Data generated for this paper	This paper	https://data.nrel.gov/submissions/184

**Software and algorithms**		

Python 3.7.4	Python Software Foundation	https://www.python.org/downloads/
ReEDSV2020	National Renewable Energy Laboratory	https://www.nrel.gov/analysis/reeds/
Emission metric algorithms	This paper	https://data.nrel.gov/submissions/184

### Resource availability

#### Lead contact

Further information and requests for resources should be directed to the lead contact, Pieter Gagnon (Pieter.Gagnon@nrel.gov).

#### Materials availability

No materials were used in this study.

### Method details

#### LRMER calculation

In this section we describe how we calculated the hourly LRMER metric. The SRMER and AER methods are described in following sections.

The LRMER was calculated with a three-step process:

1. Run a capacity expansion model that projects the operation and structural evolution of the electric grid. This is the baseline scenario.
2. Run the model again with the same inputs, but introducing a prototypical load intervention. We applied a 5% scalar increase in load across every hour, starting in 2024 (and calculated based on 2024 load) and persisting indefinitely.
3. Using the outputs of the two runs, apply the following equation.

(Equation 1) $$LRMER_{h,\;\text{levelized}}=\frac{\sum_{t=0}^{n-1}\left(\frac{E_{h,t,\;\mathrm{int}}-E_{h,t,\;\text{base}}}{{\left(1+d\right)}^{t}}\right)}{\sum_{t=0}^{n-1}\left(\frac{L_{h,t,\;\mathrm{int}}-L_{h,t,\;\text{base}}}{{\left(1+d\right)}^{t}}\right)}\;$$

Where *t* is the number of years since the introduction of the intervention, *L*_*h*,*t*,*int*_ is the end-use load within hour *h* for the intervention scenario during year *t*, and *L*_*h*,*t*,*base*_ is the same from the baseline scenario, *E*_*h*,*t*,*int*_ is the emissions during hour *h* for the intervention scenario during year *t*, and *E*_*h*,*t*,*base*_ is same from the baseline scenario, *d* is a social discount rate in real terms, and *n* is the analysis horizon in years, generally the estimated lifetime of the interventions that the metric will be applied to.

This process produces an hourly LRMER metric, levelized for a given initial year, analysis horizon, discount rate, and assumptions about the future (e.g., technology and fuel costs). This hourly metric—developed with a single prototype intervention—can then be used to estimate the impact of load interventions of varying shapes, as explained later in the *metric use and evaluation* section. We used a 3% social discount rate and a 20-year analysis horizon.

Within existing literature, this formulation is most similar to the one used by Hawkes (2014), although we include intertemporal discounting and explicitly express Equation 1 as an hourly calculation. It is also conceptually similar to the combination of “operating margin” and “build margin” as expressed in some literature (Broekhoff, 2007), although it differs by having a single term that includes both operational and structural implications of changes in electrical load, instead of estimating two margins and combining them with weights. It is also conceptually similar to the long-run source energy metric introduced for code-making in California (Energy+Environmental Economics, 2020), although the LRMER is an emissions rate whereas the Californian metric is an energy-from-depletable-fuels metric.

There are several points worth discussing with this definition of LRMER—intertemporal discounting, the treatment of storage, and phasing from SRMER into LRMER.

First, this formulation of LRMER uses intertemporal discounting at a social discount rate to produce a damages-equivalent emission rate—i.e., the emissions over the lifetime of the prototypical intervention (which can vary year-to-year) are levelized to produce a single value that, if assigned to every unit of energy over the lifetime of the intervention, would be equivalent in present-day damages to the observed emission patterns. This was done to reflect time preferences of damages—i.e., all other things being equal, it would be preferable to have damages occur later rather than sooner. If someone was to calculate aLRMER but wish to weigh the damages from emissions equally across time, the social discount rate in Equation 1 can be set to zero to reduce the present-value factor terms to 1, and therefore produce a simple averaged rate over the lifetime of the intervention. Note that the formulation of Equation 1 implicitly assumes that the marginal social cost of emissions remains constant over the lifetime of the intervention. If the marginal social costs are projected to change over the assumed lifetime, it may be appropriate to convert each year’s emissions into damages and levelize the damages themselves. Previous literature has presented long-run metrics without intertemporal discounting (Hawkes, 2014; Energy+Environmental Economics, 2020).

Secondly, prior to applying Equation 1, it is necessary to re-allocate emissions associated with the charging of storage (electric batteries and pumped-hydro storage). For our approach, we calculated the hourly net storage action for the entire nation across each year, calculated the average emission rate of non-storage generation when the storage fleet was charging on net, multiplied the energy consumed by storage charging during each hour as an estimate of the emissions induced by the storage, then allocated those emissions, weighted by generation, to the hours where storage was discharging on net.

Thirdly, as mentioned in the discussion section, it may be appropriate for some analyses to assume that the grid does not structurally adapt to an intervention immediately, if there is reason to believe the intervention would not be planned for. If an analysis calls for this, it may be appropriate to replace the estimates of the emissions prior to the structural change with an estimate produced with aSRMER method—either derived empirically or through modeling.

There are areas where this method may be improved. The treatment of energy-constrained generators (e.g., hydropower) may deserve an accounting treatment similar to storage, given the potential differences in its utilization between the baseline and intervention scenarios. The geographic disaggregation of a single model run would potentially allow for finer predictions. Portfolio standards (renewable portfolio standards and clean energy standards) are reflected in this methodology implicitly, as they are incorporated into the ReEDS model and therefore influence the generation differences between the baseline and intervention scenario, but an explicit post-processing may improve the results, as was implemented by California’s time-dependent valuation methodology for long-run source energy (Energy+Environmental Economics, 2020).

#### AER calculation

The AER metric was calculated by dividing the total emissions by total busbar generation within each hour and adjusting by a system-wide loss factor. The AER metrics were derived from the same model run that was used as the baseline model run for the LRMER calculations.

(Equation 2) $$AER_{h}=\frac{E_{h}}{G_{h\;}*\left(1-\;l\right)}$$

Where *E*_*h*,*base*_ is the emissions during hour *h*, *G*_*h*_ is the busbar level generation during hour *h*, and *l* is the system-wide loss factor.

For generation located below the busbar—only behind-the-meter PV generation in our model—the generation was adjusted by the distribution loss rate to get it into busbar-equivalent terms for this calculation. Electric batteries and pumped-hydro storage were excluded from *G*_*h*_ for the *AER* calculation, to avoid double-counting.

The system-wide loss factor includes both distribution losses and losses above the distribution system, such as transmission and storage efficiency losses. It is expressed as losses per unit of generation, where generation excludes storage technologies to avoid double-counting. We estimated the annual average distribution losses as 3.6% for the nation by subtracting modeled non-distribution losses from the eGrid Grid Gross Loss estimate, using a model run of the 2018 system and the 2018 eGriddata respectively. We assumed the distribution loss rate stayed constant across the analysis horizon. Non-distribution losses were directly calculated from the difference between pre-distribution load and generation in the model and therefore varied for every solve year of the model runs. Adjusting for system-wide losses in Equation (2) is necessary to have the *AER* metric be emissions per unit of end-use load, and therefore aligned with the other metrics analyzed in this paper.

The *AER* results shown in Figure 1 were evaluated based on the conditions in the year the intervention was introduced, whereas the *AER* results shown in Figure 2 were calculated by levelizing values with a 3% social discount rate over the same 20-year horizon as *LRMER*, applying Equation 3. The decision to use single-year values for Figure 1 was chosen to approximate the current typical practice of calculating aAER from recent empirical data.

(Equation 3) $$AER_{h,\;\text{levelized}}=\frac{\sum_{t=0}^{n-1}\left(\frac{AER_{h,t}}{{\left(1+d\right)}^{t}}\right)}{\sum_{t=0}^{n-1}\left(\frac{1}{{\left(1+d\right)}^{t}}\right)}\;$$

Where *t* is the number of years since the introduction of the intervention, *AER*_*h*,*t*_ is the *AER* during hour *h* and year *t*, *d* is a social discount rate in real terms, and _*n*_ is analysis horizon.

#### SRMER calculation

As with all the metrics calculated in this paper, the SRMER is calculated using the Augur module (Gates et al., 2021) in ReEDS, which is an hourly merit-order dispatch module with representation of storage, transmission, and minimum generation levels. As with AER, the SRMER metrics were derived from the same model run that was used as the baseline run for the LRMER calculation.

For identifying the SRMER, we first identified a specific short-run marginal generator for each hour for each of the 134 modeled balancing areas (the model’s nodes). That was done by finding each transmission-connected region (groups of regions that were connected by transmission lines whose utilization was between zero and 1 at that point in time) (Gagnon et al., 2020), and then identifying the generator with the lowest short-run marginal cost that had available capacity within that transmission-connected region, excluding energy-constrained generators (storage and hydropower). The identified generator was assumed to be the short-run marginal generator for that transmission-connected region. The emission rate of that generator, modified by an assumption of system-wide losses (as explained in the *AER calculation* section), was taken as the SRMER of an additional unit of end-use demand in any of the load nodes within that transmission-connected region at that point in time.

It should be noted that all three metric calculation methods rely on the same geographic resolution of 134 modeled balancing areas with transmission constraints between regions represented. Whereas the LRMER and AER methodologies would require additional steps to geographically disaggregate the simulation results to specific balancing areas (which was not performed for this paper), the SRMER methodology explicitly identifies aSRMER for each of the 134 balancing areas prior aggregating to the nation. Therefore, each metric is calculated using the same geographic fidelity in the model, and it is simply happenstance of the methodologies that the SRMER enables us to report and visualize region-specific values without additional calculations.

As with AER, for the results shown in Figure 1 the SRMER was evaluated based on the conditions in the year that the intervention was introduced, while in Figure 2 the SRMER was evaluated by levelizing values with a 3% social discount rate over the same 20-year horizon as LRMER, applying Equation 4. The decision to use single-year values for Figure 1 was chosen to approximate the current typical practice of calculating aSRMER from recent empirical data. Literature does exist that uses a reduced-order dispatch model to derive aSRMER from a projected system (Deetjen and Azevedo, 2019), although the approach is not common to our knowledge.

(Equation 4) $$SRMER_{h,\;\text{levelized}}=\frac{\sum_{t=0}^{n-1}\left(\frac{SRMER_{h,t}}{{\left(1+d\right)}^{t}}\right)}{\sum_{t=0}^{n-1}\left(\frac{1}{{\left(1+d\right)}^{t}}\right)}\;$$

Where *t* is the number of years since the introduction of the intervention, *SRMER*_*h*,*t*_ is the *SRMER* during hour *h* and year *t*, *d* is a social discount rate in real terms, and *n* is the analysis horizon.

This next-in-the-merit-order approach, with marginal units identified for each side of transmission congestion, has been employed by NREL in producing the publicly-available Cambium datasets (Gagnon et al., 2020). The practice of identifying a single marginal generator for a timestep and location is the same as is used for reporting marginal fuel mixtures by some system operators. For example, Monitoring Analytics—the independent market monitor for PJM interconnection—states that “marginal units are the units that set the locational marginal price in each 5-min interval. When there is congestion, there can be more than one marginal unit …” (Monitoring Analytics, 2009). Our approach can produce results that deviate from what is observed in actual markets, however, as our model does not capture all relevant operational constraints, such as maximum ramp rates and minimum up or down times.

Our SRMER approach is motivated by the same concepts, but mechanically different then the body of work that seeks to estimate a short-run marginal emission rate based off of real-time or historical operational data. Instead of identifying a specific marginal generator, some empirical approaches take the differences in total emissions divided by the difference in total generation or load between sequential hours, then use statistical techniques to derive a relationship between changes in demand and changes in system-wide emissions (Hawkes, 2010; Azevedo et al., 2020). Other organizations, such as WattTime and REsurety, are developing approaches for calculating SRMER either in real-time or retrospectively, but their methods are proprietary, prohibiting replication in this study.

Other model-based SRMER methodologies have relied on simplifying assumptions. In work for the California Energy Commission for example, a method has been employed that assumes that the short-run marginal generator is either natural gas or renewable generation (Energy+Environmental Economics, 2020). Because our analysis covers the contiguous United States, however, this simplifying assumption could not be employed.

As is shown in Supplemental Figure S2, individual regions sometimes have diurnal or seasonal trends in SRMER, although in Figure 3 we saw that the aggregation to a national weighted mean largely obscured those trends. Existing literature based on other methodologies are mixed as to the extent and strength of seasonal and diurnal trends. One study analyzed both empirical and modeled SRMER by NERC region based on 2017 data, and by examining their results we can see that four of the regions had at least a weak negative relationship between total fossil generation and SRMER—suggesting that diurnal or seasonal trends would be likely—whereas the other four regions did not have visually meaningfully correlation (Deetjen and Azevedo, 2019). A next-in-the-merit-order approach similar to the one taken in this paper, but utilizing a more sophisticated production cost model with better representations of operational constraints, observed some seasonal and diurnal trends for individual regions, but as was observed here, these trends were largely diluted when looking at the nation as a whole (Gagnon et al., 2020). Other literature saw stronger relationships between system demand and SRMER (Siler-Evans et al., 2012). Nonetheless, while different methodologies for calculating aSRMER may reveal diurnal or seasonal trends, the metric would still not capture how the structure of the grid could be influenced by a change in load, and is therefore incomplete when used for long-term planning or evaluation.

#### Metric use and evaluation

The previous three sections described how an hourly (i.e., 8760 values) version of each metric was calculated. To use one of the metrics to estimate the emissions induced by a load intervention, an 8760 load shape of the intervention was used to calculate a load-weighted emission rate for the metric (Equation 5). The AER and LRMER metrics were both calculated for the nation as a whole, whereas the SRMER metric was calculated for each of the 134 modeled balancing areas of the ReEDS model and then aggregated.

(Equation 5) $$\text{estimated emission rate}=\frac{\sum_{h=0}^{8759}\left(R_{h}*L_{h}\right)}{\sum_{h=0}^{8759}L_{h}}\;$$

Where *R*_*h*_ is the value of the emission rate metric for hour *h* and *L*_*h*_ is the end-use load of the intervention being analyzed for hour *h*.

We then ran ReEDS, our capacity expansion model, both with and without the same load intervention, introduced in 2024 and persisting indefinitely. Because the only change between each pair of runs was the load intervention, any observed difference in total emission was attributed to the intervention (Voorspools and D’haeseleer, 2000). A levelized, observed emission rate was then calculated using the same levelization formulation that was used for the LRMER calculations, also with a 20-year analysis horizon and 3% social discount rate.

The performance of each of the metrics was then evaluated by how well the estimated emission rates had anticipated the emission rates that were observed when the intervention was directly modeled.

#### Calculating implied generation mixtures

In Figure 1 we show the generation mixtures that are implied by the method used to calculate each metric. Here we explained how these implied mixtures were calculated.

The implied contribution of each fuel *f* from the AER method (*α*_*f*_) is calculated using the following equation, where *G*_*f*,*h*_ is the nation-wide generation of fuel *f* in hour *h* and *I*_*h*_ is the nation-wide load for the intervention under consideration in hour *h*.

(Equation 6) $$\alpha_{f}=\frac{\sum_{h=0}^{8759}\left(G_{f,h}*{\text{Ι}}_{h}\right)}{\sum_{h=0}^{8759}{\text{Ι}}_{h}}\;$$

The implied contribution of each fuel *f* from the SRMER method $\left(\varsigma_{f}\right)$ is calculated using the following equation, where *M*_*f*,*r*,*h*_ is a binary value that equals 1 if fuel *f* is on the short-run margin for region *r* and hour *h* and zero if not, and *I*_*r*,*h*_ is the load in region *r* and hour *h* for the intervention under consideration.

(Equation 7) $$\varsigma_{f}=\frac{\sum_{r=0}^{133}\left(\sum_{h=0}^{8759}\left(M_{f,r,h}*{Ι}_{r,h}\right)\right)}{\sum_{r=0}^{133}\left(\sum_{h=0}^{8759}\left({Ι}_{r,h}\right)\right)}\;$$

The implied contribution of each fuel *f* from the LRMER method $\left(\zeta_{f}\right)$ is calculated using the following three equations. First the difference in levelized generation by fuel for each hour is calculated, where *G*_*f*,*h*,*t*,*int*_ is the generation by fuel *f*, in hour *h*, in year *t*, from the intervention scenario, *G*_*f*,*h*,*t*,base_ is the same for the baseline scenario, n is the number of years being analyzed, and *d* is the social discount rate:

(Equation 8) $$\Psi_{f,h}=\sum_{t=0}^{n-1}\left(\frac{G_{f,h,t,\mathrm{int}}}{{\left(1+d\right)}^{t}}\right)-\sum_{t=0}^{n-1}\left(\frac{G_{f,h,t,\text{base}}}{{\left(1+d\right)}^{t}}\right)\;$$

The results of Equation 8 is then normalized by each hour’s total levelized generation difference, where i is the number of fuel types:

(Equation 9) $$\Omega_{f,h}=\frac{\Psi_{f,h}}{\sum_{f=0}^{i-1}\left(\Psi_{f,h}\right)\;}\;$$

Lastly, the normalized generation differences are weighted by the intervention’s load in each hour:

(Equation 10) $$\zeta_{f}=\frac{\sum_{h=0}^{8759}\left(\Omega_{f,h}\;*\;{Ι}_{h}\right)\;}{\sum_{h=0}^{8759}{Ι}_{h}}\;$$

#### Model and scenario description

This analysis was conducted using the Regional Energy Deployment System (ReEDS) capacity expansion model (Brown et al., 2020), using the version of the model that was used for the 2020 Standard Scenarios (Cole et al., 2020) (model tag V2020.0 in the *ReEDS_OpenAccess* repository), other than the changes necessary to introduce the load interventions. Given a set of assumptions about the future, such as fuel and technology costs, ReEDS solves a linear program to find the investment and operational decisions that minimize the overall cost of the electric system, subject to policy and operational constraints.

The ReEDS model covers the contiguous United States and has a geographic resolution of 134 load nodes connected with approximately 300 representative transmission corridors connecting the nodes. Therefore, although all of the results in the body of this paper are presented at the national level, they were derived from model runs with much finer geographic resolution.

The input assumptions used in this analysis were the same as the Mid-case from the 2020 Standard Scenarios, other than the adjustments to load in the intervention model runs. These assumptions are best understood as business-as-usual, as they assume “reference” fuel prices from the Annual Energy Outlook (EIA, 2020), “moderate” technology cost improvements from the Annual Technology Baseline (NREL, 2020), and a representation policies enacted when the study was conducted.

#### Load interventions

One of the objectives of this work was to characterize how well aLRMER could anticipate differences between load interventions that varied in what time of day they added load. To that end, we modeled a set of load interventions that focused on six time periods: day (8am to 4pm), night (10pm to 6am), morning (6am to noon), afternoon (noon to 5pm), evening (5pm to 10pm), and midday (10am to 2pm). All load interventions are defined as a percentage increase in end-use load during the year the intervention was introduced and applied based on Central time.

For each of these time-of-day focus periods, there were then 5 versions. Each version had a 5% scalar increase in load during the focus period, but varied in their load assumptions outside of the focus period.

Lastly, there was a single intervention with a 5% increase in all hours. This was the prototypical load intervention used to generate the LRMER metric for this analysis, although it was also evaluated along with the other 30 load interventions. Table shows these 31 load shapes.

**Table:** Load interventions corresponding to Figures 1 and 2, expressed as percentage of end-use load during the year the intervention is introduced, by hour of the day

	Name	1	2	3	4	5	6	7	8	9	10	11	12	13	14	15	16	17	18	19	20	21	22	23	24
1	flat	5	5	5	5	5	5	5	5	5	5	5	5	5	5	5	5	5	5	5	5	5	5	5	5
2	afternoon	0	0	0	0	0	0	0	0	0	0	0	0	5	5	5	5	5	0	0	0	0	0	0	0
3	afternoonRamp4O0	0	0	0	0	0	0	0	0	1	2	3	4	5	5	5	5	5	4	3	2	1	0	0	0
4	afternoonShoulder6	0	0	0	0	0	0	3	3	3	3	3	3	5	5	5	5	5	3	3	3	3	3	3	0
5	afternoonRamp7O1	1	1	1	1	1	2	2	3	3	4	4	5	5	5	5	5	5	5	4	4	3	3	2	2
6	afternoonRamp2O25	3	3	3	3	3	3	3	3	3	3	3	4	5	5	5	5	5	4	3	3	3	3	3	3
7	day	0	0	0	0	0	0	0	0	5	5	5	5	5	5	5	5	0	0	0	0	0	0	0	0
8	dayRamp4O0	0	0	0	0	1	2	3	4	5	5	5	5	5	5	5	5	4	3	2	1	0	0	0	0
9	dayShoulder6O0	0	0	3	3	3	3	3	3	5	5	5	5	5	5	5	5	3	3	3	3	3	3	0	0
10	dayRamp7O1	1	2	2	3	3	4	4	5	5	5	5	5	5	5	5	5	5	4	4	3	3	2	2	1
11	dayRamp2O25	3	3	3	3	3	3	3	4	5	5	5	5	5	5	5	5	4	3	3	3	3	3	3	3
12	evening	0	0	0	0	0	0	0	0	0	0	0	0	0	0	0	0	0	5	5	5	5	5	0	0
13	eveningRamp4O0	2	1	0	0	0	0	0	0	0	0	0	0	0	1	2	3	4	5	5	5	5	5	4	3
14	eveningShoulder6O0	3	3	3	3	0	0	0	0	0	0	0	3	3	3	3	3	3	5	5	5	5	5	3	3
15	eveningRamp7O1	4	3	3	2	2	1	1	1	1	1	2	2	3	3	4	4	5	5	5	5	5	5	5	4
16	eveningRamp2O25	3	3	3	3	3	3	3	3	3	3	3	3	3	3	3	3	4	5	5	5	5	5	4	3
17	midday	0	0	0	0	0	0	0	0	0	0	5	5	5	5	0	0	0	0	0	0	0	0	0	0
18	middayRamp4O0	0	0	0	0	0	0	1	2	3	4	5	5	5	5	4	3	2	1	0	0	0	0	0	0
19	middayShoulder6O0	0	0	0	3	3	3	3	3	3	3	5	5	5	5	3	3	3	3	3	3	3	0	0	0
20	middayRamp7O1	1	1	1	2	2	3	3	4	4	5	5	5	5	5	5	4	4	3	3	2	2	1	1	1
21	middayRamp2O25	3	3	3	3	3	3	3	3	3	4	5	5	5	5	4	3	3	3	3	3	3	3	3	3
22	morning	0	0	0	0	0	0	5	5	5	5	5	5	0	0	0	0	0	0	0	0	0	0	0	0
23	morningRamp4O0	0	0	1	2	3	4	5	5	5	5	5	5	4	3	2	1	0	0	0	0	0	0	0	0
24	morningShoulder6O0	3	3	3	3	3	3	5	5	5	5	5	5	3	3	3	3	3	3	3	0	0	0	0	3
25	morningRamp7O1	2	3	3	4	4	5	5	5	5	5	5	5	5	4	4	3	3	2	2	1	1	1	1	2
26	morningRamp2O25	3	3	3	3	3	4	5	5	5	5	5	5	4	3	3	3	3	3	3	3	3	3	3	3
27	night	5	5	5	5	5	5	0	0	0	0	0	0	0	0	0	0	0	0	0	0	0	0	5	5
28	nightRamp4O0	5	5	5	5	5	5	4	3	2	1	0	0	0	0	0	0	0	0	1	2	3	4	5	5
29	nightShoulder6O0	5	5	5	5	5	5	3	3	3	3	3	3	0	0	0	0	3	3	3	3	3	3	5	5
30	nightRamp7O1	5	5	5	5	5	5	5	4	4	3	3	2	2	1	1	2	2	3	3	4	4	5	5	5
31	nightRamp2O25	5	5	5	5	5	5	4	3	3	3	3	3	3	3	3	3	3	3	3	3	3	4	5	5

## Data Availability

• The datasets generated by this study have been deposited in the NREL Data Catalog: https://data.nrel.gov/submissions/184. • All original code has been deposited in the NREL Data Catalog: https://data.nrel.gov/submissions/184. • Any additional information required to reanalyze the data reported in this paper is available from the lead contact upon request.
