# Supplementary material for: Planning for the evolution of the electric grid with a long-run marginal emission rate
Source: iScience. 2022 Feb 11;25(3):103915. doi: 10.1016/j.isci.2022.103915 (PMC8873608; doi:10.1016/j.isci.2022.103915)
Supplement: Document S1. Figures S1 and S2 and Table S1 [file mmc1.pdf]

iScience, Volume *25*

Supplemental information

Planning for the evolution of the electric grid with a long-run marginal emission rate

Pieter Gagnon and Wesley Cole

Supplemental Information

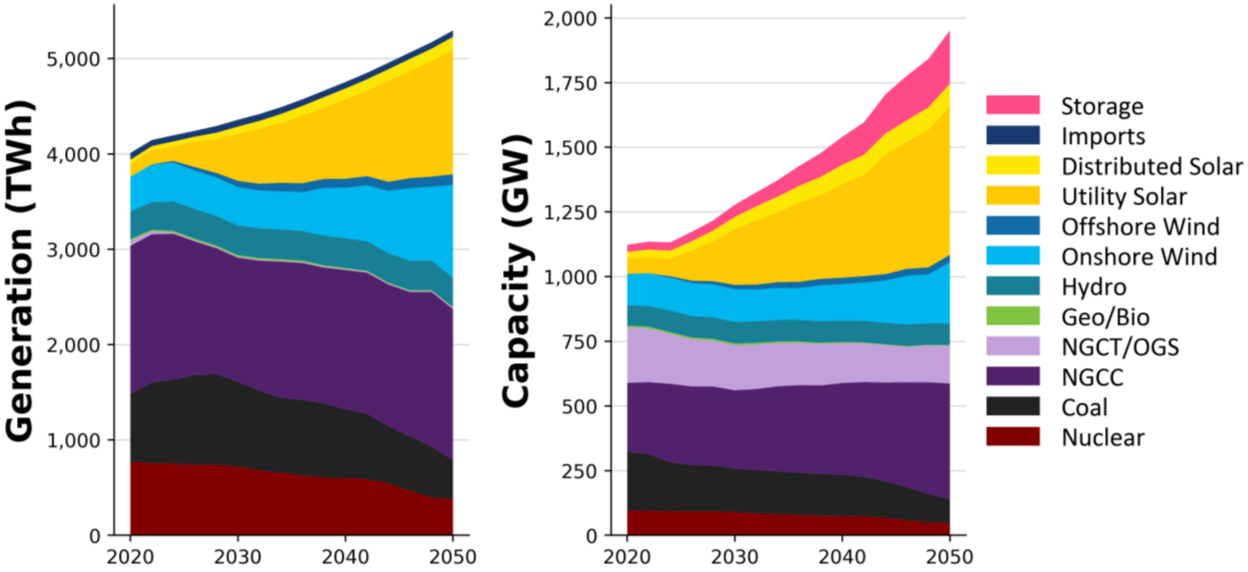

Supplemental Figure 1: U.S. power sector evolution over time for the baseline (i.e., non-intervention) model run in this study, as described in the STAR Methods. Storage generation is not shown because storage has negative net generation (due to losses). NGCC is natural gas combined cycle, NGCT is natural gas combustion turbine, OGS is oil-gas-steam, Geo/Bio is geothermal and biopower, TWh is terawatt-hours, and GW is gigawatts. Figure reproduced from the 2020 Standard Scenarios report.

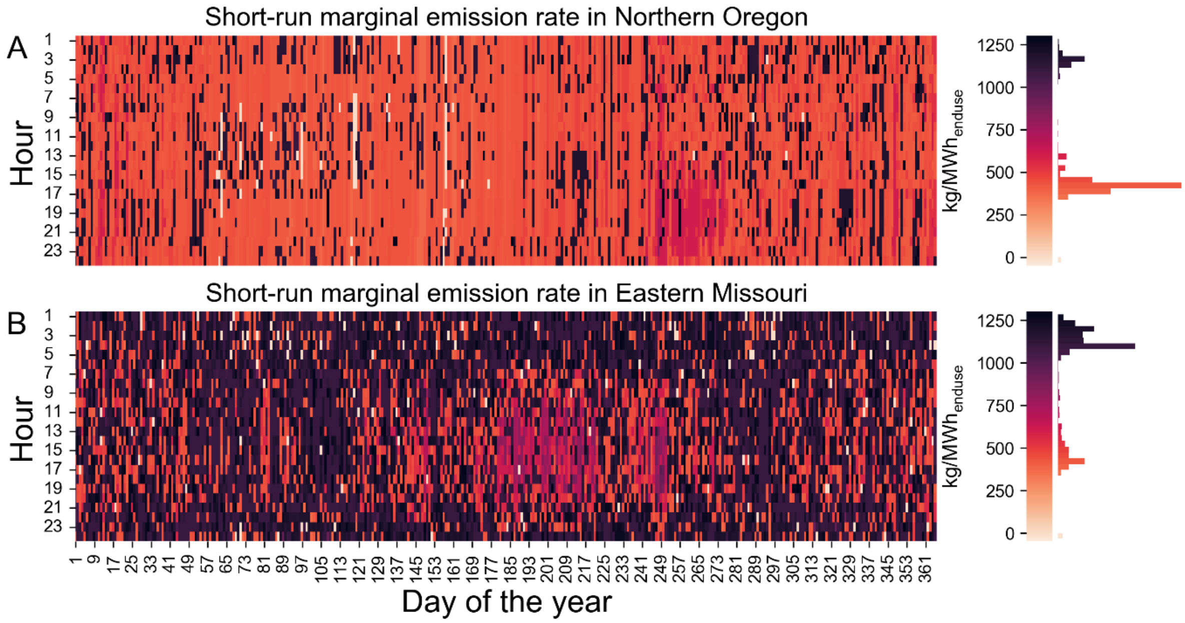

***Supplemental Figure 2: Hourly SRMER for two example regions, (A)** Northern Oregon (Pacific time zone) and **(B)** Eastern Missouri (Central time zone)**.** CO_2 from combustion per MWh of end-use load for the projected electric grid in 2024. Corresponds to the discussion of the short-run marginal emission rate derivation in the STAR Methods.*

Supplemental Table 1: Observed and estimated emission rates for the 31 load interventions evaluated in Figure 1 and Figure 2. CO_2 from combustion per MWh of end-use electric load for the contiguous United States.

	**Name**	**Observed**	**LRMER 20-year**	**SRMER 20-year**	**SRMER first-year**	**AER 20-year**	**AER first-year**
1	flat	392.1	392.1	645.3	755.5	324.4	348.2
2	afternoon	-333.8	252.6	642.8	752.3	293.2	365.6
3	afternoonRamp4O0	212.7	292.9	638.5	757.5	307.5	367.0
4	afternoonShoulder6	232.8	326.8	638.4	757.2	311.7	360.6
5	afternoonRamp7O1	315.0	344.8	640.3	757.3	316.5	358.1
6	afternoonRamp2O25	305.5	363.3	644.3	755.5	318.8	352.7
7	day	14.3	221.9	643.9	751.9	274.2	353.5
8	dayRamp4O0	215.0	283.6	640.7	755.1	296.9	357.5
9	dayShoulder6O0	328.6	324.4	642.4	756.3	307.1	354.1
10	dayRamp7O1	331.4	343.2	642.4	756.2	312.9	353.7
11	dayRamp2O25	336.2	347.3	644.4	754.7	311.7	350.4
12	evening	608.9	518.2	623.3	771.2	381.8	375.7
13	eveningRamp4O0	521.6	466.2	635.5	763.3	358.0	363.7
14	eveningShoulder6O0	498.1	439.3	640.2	759.3	345.4	357.1
15	eveningRamp7O1	401.4	423.6	642.2	758.0	338.5	354.3
16	eveningRamp2O25	411.5	415.2	641.7	758.5	335.0	353.3
17	midday	83.4	208.0	644.1	751.4	269.7	353.8
18	middayRamp4O0	105.3	240.1	642.5	752.8	281.2	355.2
19	middayShoulder6O0	258.8	304.9	640.4	756.5	304.0	357.9
20	middayRamp7O1	274.2	323.4	642.5	755.5	306.8	354.0
21	middayRamp2O25	311.9	355.7	645.1	754.8	313.6	349.3
22	morning	207.4	266.4	640.5	753.2	280.0	344.2
23	morningRamp4O0	181.0	298.5	645.5	753.4	291.5	344.6
24	morningShoulder6O0	286.0	322.7	646.8	752.4	301.1	345.5
25	morningRamp7O1	309.0	343.7	646.6	753.7	307.9	346.2
26	morningRamp2O25	385.7	364.8	644.5	755.1	314.8	347.4
27	night	638.5	525.2	668.7	748.4	346.7	316.7
28	nightRamp4O0	643.8	496.5	654.6	752.8	343.4	329.3
29	nightShoulder6O0	504.5	454.0	651.6	754.9	337.4	338.0
30	nightRamp7O1	528.8	440.3	648.6	755.0	334.1	340.7
31	nightRamp2O25	430.1	424.8	649.7	754.2	330.0	341.3
